# Conditional sparing in FLASH radiotherapy: Conformal dosimetry and ALARA remain essential

**DOI:** 10.1002/acm2.70664

**Published:** 2026-06-23

**Authors:** Chih‐Chiang Chang, Yangguang Ma, Minglei Kang, Bhudatt Paliwal, Zachary Morris, Benjamin Durkee, Chingyun Cheng

**Affiliations:** ^1^ Department of Radiation Oncology Mayo Clinic Arizona Phoenix Arizona USA; ^2^ Department of Radiation Oncology The First Affiliated Hospital of Zhengzhou University Zhengzhou Henan China; ^3^ Department of Radiation Medicine University of Wisconsin–Madison Madison Wisconsin USA; ^4^ Department of Medical Physics University of Wisconsin–Madison Madison Wisconsin USA

**Keywords:** ALARA, dosimetry, FLASH radiotherapy, normal tissue sparing, proton therapy, toxicity, treatment planning, ultrahigh dose rate

## Abstract

FLASH radiotherapy shows promise in reducing normal tissue toxicity while maintaining tumor control. However, preclinical and early clinical evidence reveal biological and technical uncertainties. We review preclinical and veterinary studies, highlight unexpected toxicities, and argue that the fundamental principles of precision and safety in radiotherapy—particularly conformal treatment and ALARA—remain crucial.

## 1 INTRODUCTION

Ultrahigh dose‐rate (UHDR) radiotherapy, also known as “FLASH” radiotherapy, has emerged as a promising treatment modality due to its potential to improve the therapeutic ratio by sparing normal tissue while preserving tumor control.^1^, ^2^, ^3^ Encouraging results obtained from preclinical studies using UHDR irradiation have demonstrated significant reductions in normal tissue damage when compared with conventional dose rate delivery, including decreased inflammation, fibrosis, and neurocognitive effects in various animal models.^4^, ^5^, ^6^, ^7^, ^8^, ^9^ These findings have motivated early clinical investigations using FLASH radiotherapy in human subjects and accelerated the development of FLASH compatible treatment platforms.^10^, ^11^, ^12^, ^13^

However, critical questions remain regarding the consistency and extent of the FLASH effect in different treatment scenarios. Recent studies have begun to explore the clinical applicability of FLASH radiotherapy (FLASH‐RT) in veterinary models.^14^, ^15^ In 2022, Borresen et al reported on a cohort of 11 dogs with oral cancers treated with electron FLASH‐RT using a single high‐dose fraction (≥ 30 Gy), outcomes demonstrated reasonable tumor control but persistent normal‐tissue toxicity.^14^ Specifically, more than one‐third of treated animals experienced severe (grade 3) acute toxicity to the oral mucosa or skin within the first month following irradiation. The toxicity rates observed in this veterinary study were comparable to those seen in canine subjects treated with conventional fractionated radiotherapy, in which severe mucositis is a common side effect. The current challenges in the clinical implementation of FLASH‐RT are further exemplified by a randomized phase III veterinary trial conducted in 2023 by Bley et al.^15^ The group investigated the efficacy and toxicity of single‐fraction (30 Gy) electron FLASH‐RT versus conventional fractionated radiotherapy (10 fractions × 4.8 Gy) in cats with early‐stage nasal planum carcinomas (T1–T2, N0).^15^ Although the tumor control in the two groups was comparable, a concerning pattern of severe late complications emerged exclusively in the FLASH‐RT cohort. Chiefly, 3 of 7 cats (43%) in the FLASH‐RT cohort developed severe maxillary bone necrosis between 9–15 months after treatment, while no cases of this toxicity were noted in the conventionally treated animals. The severe toxicity noted in the FLASH‐RT cohort was attributed by the authors to suboptimal treatment planning rather than a failure of the FLASH effect. Specifically, the administration of an ablative dose in a single fraction to cartilaginous structures violates the fundamental principles of tissue tolerance. This demonstrates that, in the context of single fraction treatments, the administration of UHDR radiation does not eliminate the need for careful optimization. In a parallel study, the same research group investigated the toxicity of a single 31 Gy electron FLASH‐RT field in mini‐pigs, focusing on the skin toxicity end point.^15^ While no acute toxicity was observed, severe late toxicity in the form of skin necrosis 7–9 months following the administration of the FLASH dose was noted, with larger irradiation volumes associated with more severe complications. These findings demonstrate that the FLASH effect has clear limitations in its ability to protect the normal tissues.

**FIGURE 1:**
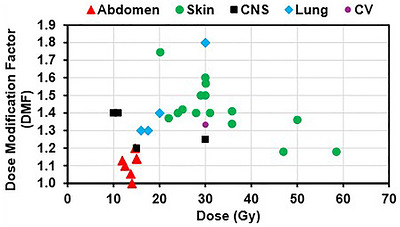
Summary of reported dose modulation factors (DMFs) and corresponding total doses across representative FLASH studies.^1^, ^4^, ^6^, ^16^, ^17^, ^18^, ^19^, ^20^, ^21^, ^22^, ^23^, ^24^, ^25^, ^26^, ^27^, ^28^, ^29^, ^30^, ^31^, ^32^, ^33^, ^34^, ^35^, ^36^, ^37^, ^38^, ^39^ CNS, central nervous system; CV, cardiovascular.

As FLASH radiotherapy advances toward broader clinical implementation, these veterinary studies carry a critical implication, that there is no guarantee against normal tissue damage, especially if basic dose‐volume constraints are violated. Organs‐at‐risk (OARs) are still at risk of radiation damage, and normal‐tissue complication probability (NTCP) is still governed by existing radiobiological principles. Even if normal‐tissue protection is observed, the degree of benefit varies considerably across preclinical studies. While some investigations report dose modifying factors suggesting ∼20%–30% tissue sparing with FLASH‐RT,^1^, ^4^, ^6^, ^16^, ^17^, ^18^, ^19^, ^20^, ^21^, ^22^, ^23^, ^24^, ^25^, ^26^, ^27^, ^28^, ^29^, ^30^, ^31^, ^32^, ^33^, ^34^, ^35^, ^36^ others demonstrate negligible or inconsistent benefit, highlighting the conditional nature of the FLASH effect^37^, ^38^, ^39^, ^40^, ^41^, ^42^, ^43^ (Figure 1). Therefore, the established framework for radiological protection, with particular emphasis on the ALARA principle (As Low As Reasonably Achievable) continues to be of major importance. Similarly, the quality of treatment planning and dose optimization for OARs is also crucial in UHDR irradiation just as in conventional radiotherapy. Until the FLASH effect can be prospectively predicted and reliably achieved across diverse clinical scenarios, conservative dose constraints must be adhered to.

## 2 CONFORMAL DOSIMETRY IS STILL IMPORTANT

While the sparing of normal tissues reported in many FLASH studies has generated excitement about its potential therapeutic applications, the persistent toxicities reported across gastrointestinal (GI), skin, lung, and CNS models indicate that FLASH cannot replace the fundamental principles of radiation protection and conformal treatment planning.^31^, ^37^, ^38^, ^39^, ^44^ Instead, these findings show that conformal dosimetry, depth management, and anatomical precision remain essential for realizing the benefits of FLASH‐RT

Preclinical FLASH platforms commonly rely on single static beams, broad unshaped fields, or minimal angular modulation, causing large regions of normal tissue to fall within the high‐dose field. For example, while FLASH has been reported to greatly decrease mortality in total abdominal irradiation (TAI), it still causes weight loss, cell depletion, and epithelial damage because of the exposure of the entire bowel to high doses of radiation.^31^ Similarly, skin studies show that FLASH reduces the severity of radiation induced skin damage but does not eliminate it. Both proton PBS and SOBP models show graded acute skin injuries, such as erythema and moist desquamation at higher doses despite the dose modifying advantage of using FLASH.^37^, ^38^ FLASH also does not uniformly spare normal tissue in the brain, as survival outcomes have been shown to be similar to conventional radiotherapy and the occurrence of dermal side effects at high doses remains similar between the two modalities.^39^

Across a broad range of preclinical studies, FLASH‐RT has been shown to reduce, rather than eliminate normal‐tissue toxicity. Limitations in beam conformality, depth‐dose geometry, and tissue specific response frequently result in residual injury. These studies taken together highlight the importance of dosimetry, precise targeting, and continued adherence to ALARA principles as FLASH‐RT moves toward clinical translation.

## 3 ADVANCES TOWARD CONFORMAL FLASH DELIVERY

The process of translating the large, uniform preclinical FLASH‐RT fields into clinically relevant geometries requires the ability to deliver highly conformal dose distributions while maintaining UHDR conditions. Several technological approaches across photon, electron and proton radiotherapy have been proposed to address this challenge, with particularly rapid development in proton therapy.^45^, ^46^, ^47^, ^48^, ^49^, ^50^, ^51^, ^52^

Protons are currently the most promising modality for conformal FLASH‐RT, mainly because of their depth dose characteristics and the potential for single energy delivery. While transmission‐beam (TB) proton FLASH‐RT has already been used clinically and easily achieves UHDR, it places the Bragg peak outside the patient, resulting in substantial exit dose and dependence on multifield optimization for conformality.^46^, ^53^ In order to achieve conformality without sacrificing the UHDR, several single‐energy proton FLASH technologies have been developed. One such technology is the Single‐Energy Bragg Peak (SEBP) technique which uses range pullback devices in conjunction with sparse spot maps. SEBP places the Bragg peak at the distal edge of the tumor and avoids energy layer switching, allowing it to maintain high beam current and eliminate exit dose.^47^, ^54^ In addition, the Single‐Energy Spread‐Out Bragg Peak (SESOBP) approach extends this capability by using ridge filters to generate an SOBP from a single energy layer enabling uniform target coverage at FLASH dose rates.^48^ Hybrid strategies that combine TB with SEBP/SESOBP modulation have also been proposed to further improve dose conformity at the edges of the target, albeit at the cost of increased delivery complexity.^49^ Finally, multi‐energy SOBP FLASH has also been suggested to improve the depth dose conformity, but maintaining UHDR across multiple energy layers is difficult with current accelerator capabilities.^50^ These methods collectively demonstrate the steady development of conformal proton FLASH‐RT, though achieving conformal dose delivery for large target volumes still has not been fully resolved.^45^

Unlike protons, conformal FLASH delivery with photons and electrons is still technologically constrained. Even with the recent implementation of MV photon FLASH, Taylor et al. found that UHDR was only possible with small, static fields (12–15 mm diameter) shaped using fixed apertures and without the capability for dynamic modulation.^51^ The authors noted, however, that the techniques used with conformal MV photon therapy, such as intensity modulation and multi‐angle beam delivery, are difficult to achieve within FLASH relevant timescales because they rely on the motion of the MLCs and gantry.^51^ Systems developed for electron FLASH applications encounter similar problems. For example, Bello et al investigated a LINAC based implementation of UHDR electron beams and found that UHDR was only achievable using fixed applicators and open fields up to 10 × 10 cm^2^, without the capability for dynamic field shaping.^54^ Additionally, the early UHDR electron beams were limited to small fields and exhibited decreasing lateral dose homogeneity with decreasing energy.^52^ These studies indicate that, at least at present, photon and electron FLASH‐RT remain constrained to static, non‐conformal geometries, again reinforcing the need for ALARA until UHDR systems capable of modulation become available.

## 4 INCONSISTENT BIOLOGICAL EVIDENCE

Although there is a significant amount of preclinical studies that report sparing of normal tissues at UHDR, such as the lung, brain, and skin,^1^, ^6^, ^16^, ^55^, ^56^, ^57^ the overall biological evidence for FLASH is still inconsistent, and depends on the model, tissues, and beam conditions. These discrepancies highlight that FLASH is not a universally reproducible phenomenon, and its manifestation is closely related to physical and biological parameters. Recently, a study titled “Whole Abdominal Pencil Beam Scanned Proton FLASH Increases Acute Lethality” reported that whole abdomen FLASH using scanned proton beams resulted in increased acute toxicity and lethality in mice when compared to conventional dose rate irradiation.^58^ This finding challenges the assumption that FLASH universally reduces toxicity and highlights the need for tissue specific evaluation. These inconsistencies suggest that the FLASH effect is highly dependent on a number of biological and dosimetric factors, including organ‐specific radiosensitivity, baseline oxygenation, dose per fraction, and pulse structure.^22^, ^28^, ^46^, ^59^, ^60^, ^61^, ^62^, ^63^ Even with similar average dose rates, variations in total dose, fractionation, and anatomical targets, FLASH‐RT deliveries can result in markedly different biological outcomes. As a result, while there is consensus that the FLASH effect is associated with a reduction in toxicity, until more robust, site specific preclinical and clinical data become available (particularly from studies conducted in large animal models or well‐structured human trials) the application of FLASH radiotherapy should be viewed with scrutiny.

## 5 INNOVATION IN RADIATION ONCOLOGY

While FLASH is fundamentally a biological effect characterized by the differential sparing of normal tissue at UHDR, it is the technology that enables FLASH to be delivered in a safe and effective manner that has the potential to transform the field of radiation therapy. Achieving the FLASH effect requires not only reexamination of radiobiological paradigms but also substantial technological advancements in beam delivery, dosimetry, and treatment planning.^45^, ^51^, ^52^, ^64^, ^65^ By pushing the boundaries of dose rate, machine performance, and system integration, the pursuit of FLASH has the potential to catalyze a new era of precision, efficiency and innovation in radiotherapy.

For example, developments in high‐throughput beam delivery systems, real‐time dose monitoring, and efficient patient setup workflows can streamline conventional radiotherapy treatments and improve clinical throughput. In parallel, as technology and infrastructure evolve to support FLASH protocols, especially in proton therapy, it may drive the development of more compact, modular, and cost‐effective systems, increasing accessibility to a wider range of treatment centers. Another promising avenue of development lies in hybrid treatment planning techniques, where FLASH is incorporated into a standard treatment course. For instance, high‐risk sub volumes or OARs could be selectively treated using FLASH boosts, with the remainder of the plan using standard IMRT or IMPT approached. These hybrid treatment plans may maximize clinical benefit while minimizing uncertainty, especially in reirradiation scenarios or in pediatric patient populations with increased long term toxicity risk.

Despite this promise, the foundation of radiotherapy must continue to be based on safety, accuracy, and patient‐centered outcomes. The principle of ALARA must continue to be an essential cornerstone of radiotherapy, particularly when considering unknowns surrounding late effects and rare toxicities. The experience with conventional radiotherapy reminds us that its full therapeutic profile—both benefits and complications—only became clear after decades of clinical use and long‐term follow‐up. FLASH radiotherapy, by contrast, is still in its early stages, and long‐term normal tissue outcomes have yet to be fully characterized. For this reason, the clinical integration of FLASH must proceed cautiously, supported by rigorous dosimetric verification, robust quality assurance, and carefully conducted clinical studies.

There is a growing consensus that advanced dosimetric tools, such as real‐time dose rate imaging and FLASH aware Monte Carlo simulations will be essential for monitoring, quantifying, and optimizing FLASH dose delivery.^64^, ^65^, ^66^, ^67^, ^68^, ^69^ Conventional QA methods and metrics may no longer be sufficient at UHDR where dose deposition and delivery occur in a matter of milliseconds and small deviations can significantly alter the delivered dose distribution. Additionally, the development of new biological dose equivalent models and FLASH specific radiobiological endpoints is critical to accurately characterize both acute and long‐term tissue responses. These technological and biological uncertainties underscore the need for continued translational research. More comprehensive animal models that better reflect human anatomy and tumor biology are urgently required, including studies in larger mammals, multiple fraction regimens, and long‐term toxicity assessment. At the same time, phase I/II clinical trials must be designed with well‐defined biological endpoints, adaptive planning, and robust toxicity monitoring, potentially incorporating advanced imaging and circulating biomarkers to track tissue response.

Finally, interdisciplinary education will be pivotal as FLASH systems are implemented in clinical environments. Physicists, physicians, dosimetrists, and therapists will need to develop a shared understanding of FLASH specific safety criteria, treatment limitations and appropriate clinical indications. This will include recognizing situations where conventional treatment approaches may still be preferred, particularly for heterogeneous tissues, large fields, and/or anatomical regions where the sparing effect of FLASH has yet to be characterized.

## 6 CONCLUSION

Ultimately, FLASH radiotherapy represents a unique combination of physics, biology, and clinical opportunity. While the benefits of FLASH radiotherapy have been identified, the effective delivery of FLASH‐RT treatments still depends on selecting appropriate treatment regimens and maintaining a high degree of dosimetric precision. Despite the current state of uncertainty regarding the robustness of the FLASH effect, the innovations it inspires in the areas of beam delivery, real‐time dosimetry, and radiobiology have the potential to drive the field of radiation therapy forward. These advancements have the potential for increasing the efficiency of radiotherapy delivery and they may also pave the way for more cost effective and accessible radiotherapy.

## AUTHOR CONTRIBUTIONS

Chih‐Chiang Chang and Yangguang Ma contributed to data curation, and writing of the original draft. Minglei Kang and Chingyun Cheng contributed to conceptualization, formal analysis, investigation, manuscript review, supervision, and project oversight. Bhudatt Paliwal, Zachary Morris, and Benjamin Durkee provided critical reviews and editing of the manuscript. All authors reviewed and approved the final version of the manuscript.

## CONFLICT OF INTEREST STATEMENT

The authors declare no conflicts of interest.

## Data Availability

The data analyzed in this study are publicly available and have been previously published in the references cited within this manuscript. No new datasets were generated during the current study.

## References

[1] Favaudon V , Caplier L , Monceau V , et al. Ultrahigh dose‐rate FLASH irradiation increases the differential response between normal and tumor tissue in mice. Sci Transl Med. 2014;6(245):245ra93. doi:10.1126/scitranslmed.3008973 10.1126/scitranslmed.3008973 25031268

[2] Romano F , Bailat C , Jorge PGç , Lerch MLF , Darafsheh A . Ultra‐high dose rate dosimetry: challenges and opportunities for FLASH radiation therapy. Med Phys. 2022;49(7):4912‐4932. doi:10.1002/mp.15649 35404484 10.1002/mp.15649 PMC9544810

[3] Vozenin M‐C , Hendry JH , Limoli C . Biological benefits of ultra‐high dose rate FLASH radiotherapy: sleeping beauty awoken. Clin Oncol. 2019;31(7):407‐415. doi:10.1016/j.clon.2019.04.001 10.1016/j.clon.2019.04.001 PMC6850216 31010708

[4] Vozenin M‐C , De Fornel P , Petersson K , et al. The advantage of FLASH radiotherapy confirmed in mini‐pig and cat‐cancer patients. Clin Cancer Res. 2019;25(1):35‐42. doi:10.1158/1078‐0432.CCR‐17‐3375 29875213 10.1158/1078-0432.CCR-17-3375

[5] Alaghband Y , Cheeks SN , Allen BD , et al. Neuroprotection of radiosensitive juvenile mice by ultra‐high dose rate FLASH irradiation. Cancers. 2020;12(6):1671. doi:10.3390/cancers12061671 32599789 10.3390/cancers12061671 PMC7352849

[6] Montay‐Gruel P , Acharya MM , Petersson K , et al. Long‐term neurocognitive benefits of FLASH radiotherapy driven by reduced reactive oxygen species. Proc Natl Acad Sci. 2019;116(22):10943‐10951. doi:10.1073/pnas.1901777116 31097580 10.1073/pnas.1901777116 PMC6561167

[7] Kim K , Kim MM , Skoufos G , et al. FLASH proton radiation therapy mitigates inflammatory and fibrotic pathways and preserves cardiac function in a preclinical mouse model of radiation‐induced heart disease. Int J Radiat Oncol Biol Phys. 2024;119(4):1234‐1247. doi:10.1016/j.ijrobp.2024.01.224 38364948 10.1016/j.ijrobp.2024.01.224 PMC11209795

[8] Kay TV , Price AL , Sprenger M , et al. Investigating the FLASH effect in a rat brain organotypic model with a novel high energy electron beam. Int J Radiat Oncol Biol Phys. 2026;124(3):759‐764. doi:10.1016/j.ijrobp.2025.09.057 PMC12713153 41077309

[9] Iturri L , Bertho Aïg , Lamirault C , et al. Proton FLASH radiation therapy and immune infiltration: evaluation in an orthotopic glioma rat model. Int J Radiat Oncol Biol Phys. 2023;116(3):655‐665. doi:10.1016/j.ijrobp.2022.12.018 36563907 10.1016/j.ijrobp.2022.12.018

[10] Vozenin M‐C , Bourhis J , Durante M . Towards clinical translation of FLASH radiotherapy. Nat Rev Clin Oncol. 2022;19(12):791‐803. doi:10.1038/s41571‐022‐00697‐z 36303024 10.1038/s41571-022-00697-z

[11] Titt U , Yang M , Wang X , et al. Design and validation of a synchrotron proton beam line for FLASH radiotherapy preclinical research experiments. Med Phys. 2022;49(1):497‐509. doi:10.1002/mp.15370 34800037 10.1002/mp.15370 PMC11931509

[12] Patriarca A , Fouillade C , Auger M , et al. Experimental set‐up for FLASH proton irradiation of small animals using a clinical system. Int J Radiat Oncol Biol Phys. 2018;102(3):619‐626. doi:10.1016/j.ijrobp.2018.06.403 30017793 10.1016/j.ijrobp.2018.06.403

[13] Mascia AE , Daugherty EC , Zhang Y , et al. Proton FLASH radiotherapy for the treatment of symptomatic bone metastases: the FAST‐01 nonrandomized trial. JAMA oncol. 2023;9(1):62‐69. doi:10.1001/jamaoncol.2022.5843 36273324 10.1001/jamaoncol.2022.5843 PMC9589460

[14] Børresen B , Arendt ML , Konradsson E , et al. Evaluation of single‐fraction high dose FLASH radiotherapy in a cohort of canine oral cancer patients. Front Oncol. 2023;13:1256760. doi:10.3389/fonc.2023.1256760 37766866 10.3389/fonc.2023.1256760 PMC10520273

[15] Rohrer Bley C , Wolf F , Gonçalves Jorge P , et al. Dose‐and volume‐limiting late toxicity of FLASH radiotherapy in cats with squamous cell carcinoma of the nasal planum and in mini pigs. Clin Cancer Res. 2022;28(17):3814‐3823. doi:10.1158/1078‐0432.CCR‐22‐0262 35421221 10.1158/1078-0432.CCR-22-0262 PMC9433962

[16] Montay‐Gruel P , Petersson K , Jaccard M , et al. Irradiation in a flash: unique sparing of memory in mice after whole brain irradiation with dose rates above 100 Gy/s. Radiother Oncol. 2017;124(3):365‐369. doi:10.1016/j.radonc.2017.05.003 28545957 10.1016/j.radonc.2017.05.003

[17] Wilson JD , Hammond EM , Higgins GS , Petersson K . Ultra‐high dose rate (FLASH) radiotherapy: silver bullet or fool's gold?. Front Oncol. 2020;9:1563. doi:10.3389/fonc.2019.01563 32010633 10.3389/fonc.2019.01563 PMC6979639

[18] Zou W , Kim H , Diffenderfer ES , et al. A phenomenological model of proton FLASH oxygen depletion effects depending on tissue vasculature and oxygen supply. Front Oncol. 2022;12:1004121. doi:10.3389/fonc.2022.1004121 36518319 10.3389/fonc.2022.1004121 PMC9742361

[19] Girdhani S , Abel E , Katsis A , et al. Abstract LB‐280: FLASH: a novel paradigm changing tumor irradiation platform that enhances therapeutic ratio by reducing normal tissue toxicity and activating immune pathways. Cancer Res. 2019;79(S13):LB‐280.

[20] Buonanno M , Grilj V , Brenner DJ . Biological effects in normal cells exposed to FLASH dose rate protons. Radiother Oncol. 2019;139:51‐55. doi:10.1016/j.radonc.2019.02.009 30850209 10.1016/j.radonc.2019.02.009 PMC6728238

[21] Khan S , Bassenne M , Wang J , et al. Multicellular spheroids as in vitro models of oxygen depletion during FLASH irradiation. Int J Radiat Oncol Biol Phys. 2021;110(3):833‐844. doi:10.1016/j.ijrobp.2021.01.050 33545301 10.1016/j.ijrobp.2021.01.050

[22] Kristensen L , Rohrer S , Hoffmann L , et al. Electron vs. proton FLASH radiation on murine skin toxicity. Radiother Oncol. 2025;206:110796. doi:10.1016/j.radonc.2025.110796 39983873 10.1016/j.radonc.2025.110796

[23] Horst F , Bodenstein E , Brand M , et al. Dose and dose rate dependence of the tissue sparing effect at ultra‐high dose rate studied for proton and electron beams using the zebrafish embryo model. Radiother Oncol. 2024;194:110197. doi:10.1016/j.radonc.2024.110197 38447870 10.1016/j.radonc.2024.110197

[24] Brown KH , Ghita‐Pettigrew M , McIvor MP , et al. Dose, dose rate and split dose impacts murine skin responses following photon FLASH irradiation. Radiother Oncol. 2025:111125. doi:10.1016/j.radonc.2025.111125 40921332 10.1016/j.radonc.2025.111125

[25] Kristensen L , Rohrer S , Johansen JG , et al. Fractionation increasingly reduces flash sparing for acute murine skin damage. Radiother Oncol. 2025;213:111209. doi:10.1016/j.radonc.2025.111209. Available at SSRN 5293782. 41110805 10.1016/j.radonc.2025.111209

[26] Bogaerts E , Macaeva E , Qamhiyeh S , et al. FLASH or flare: variable intestinal toxicity results in a mouse model following proton pencil beam scanning irradiation on a clinical superconducting synchrocyclotron. bioRxiv. 2025. doi:2025.09.17.676536

[27] Kristensen L , Overgaard C , Johansen J , et al. Fixation method influences FLASH skin sparing in an in vivo leg model. Acta Oncol. 2025;64:43972. doi:10.2340/1651‐226X.2025.43972 40762144 10.2340/1651-226X.2025.43972 PMC12340987

[28] Sesink A , Geyer R , Devanand P , et al. Decrease in dose per fraction impairs the FLASH sparing effect in murine intestine model. Radiother Oncol. 2026;214:111262. doi:10.1016/j.radonc.2025.111262 41192770

[29] Ilina A , Thomas WS , Cao Xu , et al. FLASH effect is diminished by daily fractionation of electron RT in mouse skin. Phys Med Biol. 2025;70(23):235020. doi:10.1088/1361‐6560/ae205e 10.1088/1361-6560/ae205e PMC12647958 41248557

[30] Sesink A , Soutter L , Geyer RW , Böhlen TT , Bailat C , Grilj V . Impact of increased oxygen concentration on the FLASH sparing effect in mice is tissue dependent. British Journal of Radiology. 2026;99(1178):254–262. 41339268 10.1093/bjr/tqaf290

[31] Levy K , Natarajan S , Wang J , et al. Abdominal FLASH irradiation reduces radiation‐induced gastrointestinal toxicity for the treatment of ovarian cancer in mice. Sci Rep. 2020. 10(1):21600. doi:10.1038/s41598‐020‐78017‐7 33303827 10.1038/s41598-020-78017-7 PMC7728763

[32] Loo BW , Schuler E , Lartey FM , et al. (P003) delivery of ultra‐rapid flash radiation therapy and demonstration of normal tissue sparing after abdominal irradiation of mice. Int J Radiat Oncol Biol Phys. 2017. 98(2):E16. doi:10.1016/j.ijrobp.2017.02.101

[33] Ruan J‐L , Lee C , Wouters S , et al. Irradiation at ultra‐high (FLASH) dose rates reduces acute normal tissue toxicity in the mouse gastrointestinal system. Int J Radiat Oncol Biol Phys. 2021. 111(5):1250‐1261. doi:10.1016/j.ijrobp.2021.08.004 34400268 10.1016/j.ijrobp.2021.08.004 PMC7612009

[34] Field, S , Bewley D . Effects of dose‐rate on the radiation response of rat skin. Int J Radiat Oncol Biol Phys, Chem Med. 1974. 26(3):259‐267. 10.1080/09553007414551221 4547756

[35] Hendry JH , Moore JV , Hodgson BW , Keene JP . The constant low oxygen concentration in all the target cells for mouse tail radionecrosis. Radiat Res. 1982. 92(1):172‐181. doi:10.2307/3575852 7134382

[36] Hornsey, S , Bewley D . Hypoxia in mouse intestine induced by electron irradiation at high dose‐rates. Int J Radiat Biol Relat Stud Phys Chem Med. 1971. 19(5):479‐483. 5314348 10.1080/09553007114550611

[37] Kristensen L , Poulsen PR , Kanouta E , et al. Spread‐out Bragg peak FLASH: quantifying normal tissue toxicity in a murine model. Front Oncol. 2024. 14:1427667. doi:10.3389/fonc.2024.1427667 39026976 10.3389/fonc.2024.1427667 PMC11256197

[38] Sørensen, BS , Krzysztof MS , Ankjærgaard C , et al. In vivo validation and tissue sparing factor for acute damage of pencil beam scanning proton FLASH. Radiother Oncol. 2022. 167:109‐115. 34953933 10.1016/j.radonc.2021.12.022

[39] Liljedahl E , Konradsson E , Linderfalk K , et al. Comparable survival in rats with intracranial glioblastoma irradiated with single‐fraction conventional radiotherapy or FLASH radiotherapy. Front Oncol. 2024. 13:1309174. doi:10.3389/fonc.2023.1309174 38322292 10.3389/fonc.2023.1309174 PMC10845047

[40] Cuitiño, MC , Fleming JS , Jain S , et al. Comparison of gonadal toxicity of single‐fraction ultra‐high dose rate and conventional radiation in mice. Adv Radiat Oncol. 2023. 8(4):101201. 37008254 10.1016/j.adro.2023.101201 PMC10050676

[41] Zhang Q , Gerweck LE , Cascio E , et al. Absence of tissue‐sparing effects in partial proton FLASH irradiation in murine intestine. Cancers. 2023. 15(8):2269. doi:10.3390/cancers15082269 37190197 10.3390/cancers15082269 PMC10137009

[42] Melemenidis, S , Viswanathan V , Dutt S , et al. Effectiveness of FLASH vs. conventional dose rate radiotherapy in a model of orthotopic, murine breast cancer cancers. Cancers. 2025. 17(7):1095. 40227580 10.3390/cancers17071095 PMC11988084

[43] Williams MT , Regan SL , Fritz AL , et al. Effects of whole brain proton irradiation at conventional or ultra‐high dose rate (FLASH), in adult male Sprague Dawley rats. Sci Rep. 2025. 15(1):10602. doi:10.1038/s41598‐025‐94534‐9 40148391 10.1038/s41598-025-94534-9 PMC11950509

[44] Gjaldbæk BW , Arendt ML , Konradsson E , et al. Long‐term toxicity and efficacy of FLASH radiotherapy in dogs with superficial malignant tumors. Front Oncol. 2024. 14:1425240. doi:10.3389/fonc.2024.1425240 39077466 10.3389/fonc.2024.1425240 PMC11284943

[45] Ma, Y , Zhang T , Selvaraj B , et al., Advancing proton FLASH radiation therapy: innovations, techniques, and clinical potentials. Int J Radiat Oncol Biol Phys. 2025;123(3):876‐890. doi:10.1016/j.ijrobp.2025.05.076 40513679

[46] Kang M , Wei S , Choi JI , Simone CB , Lin H . Quantitative assessment of 3D dose rate for proton pencil beam scanning FLASH radiotherapy and its application for lung hypofractionation treatment planning. Cancers. 2021. 13(14):3549. doi:10.3390/cancers13143549 34298762 10.3390/cancers13143549 PMC8303986

[47] Kang M , Wei S , Choi JI , Lin H , Simone CB . A universal range shifter and range compensator can enable proton pencil beam scanning single‐energy Bragg peak FLASH‐RT treatment using current commercially available proton systems. Int J Radiat Oncol Biol Phys. 2022. 113(1):203‐213. doi:10.1016/j.ijrobp.2022.01.009 35101597 10.1016/j.ijrobp.2022.01.009

[48] Liu R , Charyyev S , Wahl N , et al. An integrated physical optimization framework for proton stereotactic body radiation therapy FLASH treatment planning allows dose, dose rate, and linear energy transfer optimization using patient‐specific ridge filters. Int J Radiat Oncol Biol Phys. 2023. 116(4):949‐959. doi:10.1016/j.ijrobp.2023.01.048 36736634 10.1016/j.ijrobp.2023.01.048

[49] Ma C , Yang X , Chang C‐W , et al. Feasibility study of hybrid inverse planning with transmission beams and single‐energy spread‐out Bragg peaks for proton FLASH radiotherapy. Med Phys. 2023. 50(6):3687‐3700. doi:10.1002/mp.16370 36932635 10.1002/mp.16370 PMC11700378

[50] Zeng Y , Li H , Wang W , et al. Feasibility study of multiple‐energy Bragg peak proton FLASH on a superconducting gantry with large momentum acceptance. Med Phys. 2024. 51(3):2164‐2174. doi:10.1002/mp.16932 38169535 10.1002/mp.16932

[51] Taylor ERJF , Tullis IDC , Vojnovic B , Petersson K . Megavoltage photon FLASH for preclinical experiments. Med Phys. 2025;52(7):e17891. doi:10.1002/mp.17891 40387520 10.1002/mp.17891 PMC12258002

[52] Dal Bello R , von der Grün J , Fabiano S , et al. Enabling ultra‐high dose rate electron beams at a clinical linear accelerator for isocentric treatments. Radiother Oncol. 2023. 187:109822. 37516362 10.1016/j.radonc.2023.109822

[53] Van De Water, S , Safai S , Schippers JM , et al. Towards FLASH proton therapy: the impact of treatment planning and machine characteristics on achievable dose rates. Acta Oncol. 2019. 58(10):1463‐1469. 31241377 10.1080/0284186X.2019.1627416

[54] Lattery G , Kaulfers T , Cheng C , et al. Pencil beam scanning Bragg peak FLASH technique for ultra‐high dose rate intensity‐modulated proton therapy in early‐stage breast cancer treatment. Cancers. 2023. 15(18):4560. doi:10.3390/cancers15184560 37760528 10.3390/cancers15184560 PMC10527307

[55] Bourhis J , Sozzi WJ , Jorge PGç , et al. Treatment of a first patient with FLASH‐radiotherapy. Radiother Oncol. 2019. 139:18‐22. doi:10.1016/j.radonc.2019.06.019 31303340 10.1016/j.radonc.2019.06.019

[56] Fouillade C , Curras‐Alonso S , Giuranno L , et al. FLASH irradiation spares lung progenitor cells and limits the incidence of radio‐induced senescence. Clin Cancer Res. 2020. 26(6):1497‐1506. doi:10.1158/1078‐0432.CCR‐19‐1440 31796518 10.1158/1078-0432.CCR-19-1440

[57] Sørensen, BS , Sitarz MK , Ankjærgaard C , et al. Pencil beam scanning proton FLASH maintains tumor control while normal tissue damage is reduced in a mouse model. Radiother Oncology. 2022. 175:178‐184. 10.1016/j.radonc.2022.05.014 35595175

[58] Bell BI , Velten C , Pennock M , et al. Whole abdominal pencil beam scanned proton FLASH increases acute lethality. Int J Radiat Oncol Biol Phys. 2025. 121(2):493‐505. doi:10.1016/j.ijrobp.2024.09.006 39299552 10.1016/j.ijrobp.2024.09.006 PMC12142639

[59] Sørensen, BS , Kanouta E , Ankjærgaard C , et al. Proton FLASH: impact of dose rate and split dose on acute skin toxicity in a murine model. Int J Radiat Oncol Biol Phys. 2024. 120(1):265‐275. 38750904 10.1016/j.ijrobp.2024.04.071

[60] Friedl AA , Prise KM , Butterworth KT , Montay‐Gruel P , Favaudon V . Radiobiology of the FLASH effect. Med Phys. 2022. 49(3):1993‐2013. doi:10.1002/mp.15184 34426981 10.1002/mp.15184

[61] Hunter, DI , Sunnerberg JP , Tavakkoli AD , et al. Intermediate tissue oxygen level is required to observe murine FLASH skin sparing. bioRxiv. 2025. doi:10.1101/2025.10.06.680759

[62] Bedford, JL . Pulse‐by‐pulse treatment planning and its application to generic observations of ultra‐high dose rate (FLASH) radiotherapy with photons and protons. Phys Med Biol. 2025. 70(4):045010. doi:10.1088/1361‐6560/adaf04 10.1088/1361-6560/adaf04 39870031

[63] Sunnerberg, JP , Hunter DI , Sloop AM , et al. Timescale of FLASH sparing effect determined by varying temporal split of dose delivery in mice. Int J Radiat Oncol Biol Phys. 2026;124(3):831–841. 41046060 10.1016/j.ijrobp.2025.09.052 PMC13373776

[64] Bookbinder A , Selvaraj B , Zhao X , et al. Validation and reproducibility of in vivo dosimetry for pencil beam scanned FLASH proton treatment in mice. Radiother Oncol. 2024. 198:110404. doi:10.1016/j.radonc.2024.110404 38942121 10.1016/j.radonc.2024.110404

[65] Chang C‐C , Selvaraj B , Zhao X , et al. First experience with third‐party validations: a robust calibration and QA procedure for proton FLASH delivery. J Appl Clin Med Phys. 2025. 26(10):e70269. doi:10.1002/acm2.70269 41014172 10.1002/acm2.70269 PMC12475972

[66] Diffenderfer ES , Verginadis II , Kim MM , et al. Design, implementation, and in vivo validation of a novel proton FLASH radiation therapy system. Int J Radiat Oncol Biol Phys. 2020. 106(2):440‐448. doi:10.1016/j.ijrobp.2019.10.049 31928642 10.1016/j.ijrobp.2019.10.049 PMC7325740

[67] Jung S , Kim InJ , Yi C‐Y , et al. Electron and proton FLASH beam dosimetry using unified alanine, EBT‐XD, and HD‐V2 Gafchromic film dosimeters. Med Phys. 2025. 52(10):e70022. doi:10.1002/mp.70022 40999628 10.1002/mp.70022 PMC12464357

[68] Wei S , Lin H , Cheng C , Choi JI , Simone CB , Kang M . An ultra‐high dose rate Bragg peak tracking technique provides more affordable proton radiotherapy for cancer patients: from principle to experimental validation. Radiother Oncol. 2025. 206:110800. doi:10.1016/j.radonc.2025.110800 39988304 10.1016/j.radonc.2025.110800

[69] Guo L , Zhou B , Tsai Yi‐C , Jiang K , Iakovenko V , Wang KK‐H . Comprehensive characterization and validation of a fast‐resolving (1000 Hz) plastic scintillator for ultra‐high dose rate electron dosimetry. Med Phys. 2025. 52(10):e70006. doi:10.1002/mp.70006 40983874 10.1002/mp.70006 PMC12454735

